# Alzheimer’s disease: an axonal injury disease?

**DOI:** 10.3389/fnagi.2023.1264448

**Published:** 2023-10-19

**Authors:** Liang Dan, Zhaohui Zhang

**Affiliations:** Department of Neurology, Renmin Hospital of Wuhan University, Wuhan, Hubei, China

**Keywords:** Alzheimer’s disease, axon, tau, amyloid-beta, APOE, TREM2

## Abstract

Alzheimer’s disease (AD) is the primary cause of dementia and is anticipated to impose a substantial economic burden in the future. Over a significant period, the widely accepted amyloid cascade hypothesis has guided research efforts, and the recent FDA approval of an anti- amyloid-beta (Aβ) protofibrils antibody, believed to decelerate AD progression, has further solidified its significance. However, the excessive emphasis placed on the amyloid cascade hypothesis has overshadowed the physiological nature of Aβ and tau proteins within axons. Axons, specialized neuronal structures, sustain damage during the early stages of AD, exerting a pivotal influence on disease progression. In this review, we present a comprehensive summary of the relationship between axonal damage and AD pathology, amalgamating the physiological roles of Aβ and tau proteins, along with the impact of AD risk genes such as APOE and TREM2. Furthermore, we underscore the exceptional significance of axonal damage in the context of AD.

## 1. Introduction

AD was initially described in 1906 by German neuropathologist Alois Alzheimer. It is a neurodegenerative disorder known for its progressive cognitive and behavioral impairments, including the inability to form new memories and the loss of important ones (Koffie et al., 2011). Over time, AD leads to a substantial decline in executive functions, ultimately preventing patients from independently performing daily activities (Scheff et al., 2014). Presently, AD affects over 50 million individuals worldwide (Roy et al., 2023) and the number continues to escalate and is projected to double by 2050 (Velazquez et al., 2018). With advances in healthcare contributing to longer lifespans, the prevalence of AD, primarily affecting the elderly, is also on the rise (Kashyap et al., 2019). Given the absence of current treatments to prevent or reverse AD, the cumulative costs of caring for AD patients will increasingly burden the global healthcare system in the years ahead (Forner et al., 2017).

AD is characterized by a continuum process involving extracellular amyloid deposition, tau tangles in neuronal cells, alterations in neural networks, and neuronal loss. The recent FDA approval of the monoclonal antibody Lecanemab, which exhibits a high affinity for Aβ protofibrils (van Dyck et al., 2023), holds promise for its application in AD treatment (Larkin, 2023). In-depth genome-wide association studies (GWAS) have significantly contributed to the understanding of sporadic AD (sAD). For instance, Jansen et al. conducted a case–control study with over 600,000 cases, identifying 29 AD risk loci through genome-wide meta-analysis (Jansen et al., 2019). Moreover, Kunkle et al. analyzed genomes of more than 90,000 individuals, including over 30,000 LOAD cases, and discovered 25 genome-wide significant AD risk loci. Pathway analysis revealed involvement in immunity, lipid metabolism, tau protein, and amyloid precursor protein (APP) metabolism (Kunkle et al., 2019). These GWAS studies emphasize the importance of genes such as APOE and TREM2 in sAD.

As the most complex cells in the human body, neurons possess distinctive structures including soma, axons, and dendrites. In the nervous system, axons are so extensive that up to 99% of neuronal cytoplasm may be contained within axons in the peripheral nervous system. Axons serve critical functions, chiefly the conduction of electrical signaling and support of synaptic material and energy. Axons can integrate thousands of inputs (i.e., synaptic afferents) into a single output (i.e., action potential). Nevertheless, the role of axons is not limited solely to transmitting action potentials from the initiation site at the soma to the terminus. Axons have been proposed to possess computational capacity and implement various complex operations that ensure appropriate signaling. The biophysical properties of voltage-gated channels together with the geometric architecture of axons determine the propagation timing of output messages in different axonal branches. Moreover, local shaping of axonal action potentials may subsequently dictate synaptic efficacy during repetitive stimulation. This emphasizes the intricacy of axons and their additional roles in neural communication [summarized in review (Debanne et al., 2011)]. Importantly, uninterrupted material transport and energy delivery within axons are imperative to sustain unbroken electrical signaling. Specialized axonal structures including the initial segment, nodes of Ranvier, myelin sheath, and interactions with oligodendrocytes and microglia play vital roles. The multifaceted signaling and material/energy transfer signify that axonal function is vulnerable to disruption and may have pivotal involvement in pathological processes.

Although axonal degeneration may be a common lesion in neurodegenerative diseases, and axonal injury occurs in the early stages of AD (Salvadores et al., 2017), axonal pathology is still underappreciated in AD compared to synapses. In this study, we build upon previous research and integrate recent evidence to underscore the significance of axonal function in AD. In our review, we begin by discussing the physiological functions of Tau and amyloid beta (Aβ) within axons, the axonal milieu and tau aggregation, and the bidirectional relationship between axonal injury and the pathology of Aβ/tau. Next, we discuss the role of axonal injury in neuronal structural changes, emphasizing that axonal injury occurs early in Alzheimer’s disease (AD). Additionally, we summarize the vulnerabilities axons face during aging and the effects of AD risk genes on axons. Finally, we conclude by summarizing the current prospects for microtubule stabilizers. In summary, our review underscores the critical role of axonal injury in AD.

## 2. Aβ and tau are important components of the axonal system

The high conservation of the protein sequences of Aβ and tau suggests significant physiological functions for both proteins. Aβ, an ancient neuropeptide, is remarkably conserved across vertebrates, with the human Aβ sequence shared by 60–70% of vertebrate species (Tharp and Sarkar, 2013). Tau, on the other hand, is an axonal microtubule-binding protein highly conserved in mammalian species. Evidence of MAPT-like genes has been discovered even in lampreys, hagfish, and sharks, indicating an origin dating back 550 million years (Sündermann et al., 2016). The predominant expression and critical physiological functions of both tau and Aβ within axons imply an association between these two proteins and axonal pathology.

### 2.1. The effects of Aβ on axon

Aβ is generated through the cleavage of APP, a transmembrane glycoprotein with a molecular weight ranging from 100 to 140 kDa. APP is widely expressed in brain neurons, particularly at the synaptic level, as well as in blood cells (including platelets), vessels, and to a lesser extent, astrocytes (van der Kant et al., 2020). The cleavage of full-length APP by α-secretase and β-secretase releases soluble ectodomains (sAPPα and sAPPβ) while generating C-terminal fragments (CTFs) composed of transmembrane and short cytoplasmic domains (C83 and C99, respectively). Subsequently, γ-secretase cleaves APP-CTFs to release the APP intracellular domain (AICD) and generate extracellular p3 or Aβ peptides, with Aβ ranging from 38 to 43 amino acids in length. In summary, the generation of Aβ peptides occurs through either the α-secretase-mediated non-amyloidogenic pathway or the β-secretase-mediated amyloidogenic pathway (Deyts et al., 2016; van der Kant et al., 2020).

It is important to acknowledge that APP and its products have normal and crucial physiological roles in neurons. In mammals, APP belongs to a small gene family that includes APP-like proteins 1 and 2 (APLP1 and APLP2) encoded by distinct genes (Shariati and De Strooper, 2013). Within axons, they are primarily transported in vesicles and become enriched in active areas. Interestingly, surface accumulation of APP favors non-amyloidogenic processing, while retention of APP in acidic compartments (e.g., early endosomes) promotes amyloidogenic processing (Müller et al., 2017). Moreover, soluble proteins interacting with APP include netrin, F-spondin, and pancortins, and all these proteins are implicated in neuronal migration and differentiation (Müller et al., 2017). Axonal connectivity in APP^−/−^ mice is only mildly impaired: smaller forebrain commissures (Magara et al., 1999) and rather subtle roles in the retinotectal system have been described (Osterhout et al., 2015). Compared to wildtype mice, APP^−/−^ mice exhibit significantly increased injury and functional deficits after mild TBI, which can be rescued by recombinant APPsα (Corrigan et al., 2014). Besides, experiments in APP knockout mice have shown that sAPPα rather than sAPPβ can restore deficits in spine density, long-term potentiation, and spatial learning (Hick et al., 2015; Richter et al., 2018) underscoring the importance of APP proteolytic pathways.

Despite the potential for Aβ abnormal aggregation and cytotoxicity, Aβ also has various important functions (van der Kant et al., 2020). For example, it can trigger or maintain intracellular signaling pathways essential for neurotransmission, including regulating excitation/inhibition balance (Abramov et al., 2009) and synaptic vesicle transport (Park et al., 2013a). Moreover, Aβ released in the synaptic cleft plays a key role in maintaining neuronal bioenergetic levels (Giuffrida et al., 2015). In summary, APP and its cleavage products have significant roles within axons.

### 2.2. The effects of tau on axon

Tau, a microtubule-binding protein (MTBP), is predominantly present in axons and accumulates at the distal unstable end (Baas and Qiang, 2019). In its precursor messenger RNA (pre-mRNA), alternative splicing of exon 2 (E2), E3, and E10 generates six tau isoforms, and the length range of tau protein is between 352 and 441 amino acids (Götz et al., 2019). Depending on the existence of three or four repeats of MTBDs (microtubule binding domains), tau can be classified into 3R tau (exon 10 exclusion) and 4R tau (exon 10 inclusion) (Sexton et al., 2022). The levels of the 3R and 4R isoforms are approximately equal in the adult human brain (Strang et al., 2019). Interestingly, AD exhibits equivalent levels of 3R and 4R tau as well as regionally disproportionate tau isoforms (Dugger and Dickson, 2017). The relative differences in tau isoform ratios may account for distinct propagation patterns of tau (Boyarko and Hook, 2021).

It is widely recognized that tau plays a critical role in stabilizing microtubules (MTs), which undergo dynamic instability and transition between growth, shrinkage, and pause phases referred to as catastrophe and rescue, respectively. In order to examine the impact of tau depletion on axonal MT stability, Qiang et al. employed pan-tau-subtype RNA interference in cultured rat cortical or sympathetic neurons (Qiang et al., 2018). Following 4 days of tau depletion, there were no observable changes in axonal length or morphology compared to control conditions, but the MT mass per unit axonal length was reduced. Moreover, tau-depleted axons exhibited elevated levels of Glu-tubulin per unit axonal length, indicating the presence of more stable MT domains. Conversely, the abundance of unstable Tyr-tubulin-rich domains was significantly reduced in tau-depleted axons compared to control axons (Qiang et al., 2018). Therefore, rather than destabilizing stable MT domains, tau depletion resulted in the partial loss of unstable MT domains (Qiang et al., 2018). However, another study showed that tau recruits end-binding proteins (EBs) to stable microtubule bundles, preventing their tracking to microtubule ends and increasing catastrophe frequency (Ramirez-Rios et al., 2016). The absence of tau is consistent with the reduced density of MTs, and the specific morphology of tau binding to MTs in axonal repair may suggest a close relationship between tau and the stability of MT (Volkov and Akhmanova, 2023). In the future, additional studies are needed to explore the role of tau in regulating MTs.

Despite this, tau still plays important physiological roles (Limorenko and Lashuel, 2022). Tau knockdown has been shown to impair growth cone repulsion (Li et al., 2014; Biswas and Kalil, 2018), disrupt axonal extension (Caceres and Kosik, 1990), delay neuronal maturation (Caceres and Kosik, 1990; Dawson et al., 2001), and reduce microtubule density (Harada et al., 1994). Moreover, tau depletion has been associated with reduced action potential firing and altered excitatory/inhibitory ratio in pyramidal cells of acute cortical slices (DeVos et al., 2013; Hall et al., 2015; Chang et al., 2021). Tau also plays a role in spatially regulating the balance of microtubule-dependent axonal transport (Dixit et al., 2008). In the presynaptic terminal, pathogenic tau binds to synaptic vesicles through its N-terminal structural domain and interferes with presynaptic processes, including synaptic vesicle mobility and release rate, ultimately leading to reduced neurotransmission in fly and rat neurons (Zhou et al., 2017). Additionally, tau is associated with the disruption of the presynaptic vesicle pool (McInnes et al., 2018; Largo-Barrientos et al., 2021). Tau binding to the synaptic vesicle protein synaptophysin-3 restricts synaptic vesicle movement and impairs neurotransmission in tauopathy models. Furthermore, there is evidence of direct interactions between tau and various synaptic proteins, including β-synuclein, synaptophysin-3, synaptotagmin, synapsin-1B, synaptogyrin, and synaptotagmin-1 (Liu et al., 2016; McInnes et al., 2018; Largo-Barrientos et al., 2021).

An intriguing finding in patients with frontotemporal dementia (FTD) is the presence of partial deletions in the MAPT gene, leading to truncated tau proteins that lack the first microtubule-binding repeat (Rovelet-Lecrux et al., 2009). These truncated tau proteins show a substantial decrease in microtubule-binding ability while gaining the capacity to sequester MAP 1B. This may result in both loss-of-function and gain-of-function disturbances in microtubule dynamics, akin to double knockout mice (Rovelet-Lecrux et al., 2009). Importantly, tau protein levels are reduced in FTD (Papegaey et al., 2016), intellectual disability (Mazzon et al., 2018), Parkinson’s disease (PD) (Lei et al., 2012), normal aging (Mukaetova-Ladinska et al., 1996) and AD (Nieto et al., 1989). Abnormal modifications and aggregation of tau may lower its concentration, and this physiological deficiency of tau may also be part of the mechanism of neurodegeneration (Kent et al., 2020).

Surprisingly, tau knockout strains do not display obvious phenotypic abnormalities (DeVos et al., 2013), and the nervous systems of tau-deficient mice appear normal immunohistochemically (Harada et al., 1994). Recent studies have also shown that tau gene deletion does not affect axon regeneration or retinal neuron survival in the injured mouse visual system (Rodriguez et al., 2020). It is possible that compensation occurs between the three genes of the MAP 2/Tau family expressed in vertebrates, which correspond to the proteins MAP 2, MAP 4, and Tau (Dehmelt and Halpain, 2005). Similarly to tau knockout, MAP 2 knockout mice do not exhibit major defects in brain morphology, but they do show a reduction in microtubule density in dendrites. Additionally, cultured neurons with MAP 2 knockout exhibit decreased dendritic length, indicating the role of MAP 2 in supporting dendritic elongation (Harada et al., 2002). Interestingly, the structurally unrelated microtubule-associated protein MAP 1B seems to have some redundancy with both Tau (Takei et al., 2000) and MAP 2 (Teng et al., 2001). In fact, the mortality rate of MAPT and MAP 1B double knockout mice is high, demonstrating a synergistic disruption of growth cone dynamics, axonal extension, and neuronal migration. This results in axonal bundle defects and abnormal neuronal layer formation (Takei et al., 2000). Moreover, the expression of microtubule-associated protein 1A (MAP 1A) increases significantly in mice lacking tau protein, potentially compensating for the loss of tau (Harada et al., 1994). While no changes in MAP 1B expression levels have been found, another study showed that crossing tau mutants with MAP 1B knockout mice exacerbates axonal bundle dysplasia, neuronal layering disorders, and impaired primary neuron maturation observed in MAP 1B knockout mice (Takei et al., 2000). *Drosophila melanogaster* has been employed as an ideal model for studying the physiological role of tau since Tau is the only member of the MAP 2/Tau family in this organism (Dehmelt and Halpain, 2005). Studies on Drosophila tau mutants have revealed changes in microtubule organization and reduced microtubule density in adult brain axons (Bolkan and Kretzschmar, 2014), although the mt number did not change in oocytes, indicating histological differences. Additionally, the loss of tau in Drosophila tauKO resulted in phenotypic and behavioral changes, highlighting a new role of Tau in regulating synapse formation and maintenance during aging by coordinating the intracellular transport of synaptic proteins (Burnouf et al., 2016). This role of Tau in synaptic regulation overlaps with the function of spectraplakin Short Stop (Shot), a large actin-MT linker molecule and potent regulator of MT (Voelzmann et al., 2016). Depletion of Tau resulted in a reduced number and density of microtubules within axons, whereas Tau overexpression had the opposite effect. Furthermore, analysis of vesicle transport in tau mutants revealed changes in vesicle mobility without altering the total number of moving vesicles, while both aspects were affected when Tau was overexpressed (Talmat-Amar et al., 2018).

Behavioral deficits and motor deficits develop in tau knockout mice only when aged (Lei et al., 2012; Ma et al., 2014). Aged tau knockout mice (OKO), but not middle-aged tau knockout mice (MKO), exhibit Morris water maze deficits and loss of acetylated α-microtubulin and excitatory synaptic proteins in the hippocampus. Mild motor deficits and reduced tyrosine hydroxylase (TH) levels in the substantia nigra are observed in middle-aged mice. Deletion of tau leads to elevated levels of MAP 1A, MAP 1B, and MAP 2 in MKO mice, followed by the loss of MAP 2 and MAP 1B in OKO mice. Intriguingly, dietary supplementation with docosahexaenoic acid (DHA) or a combination of DHA and alpha-linolenic acid (ALA) partially restores hippocampal synaptic deficits and TH reduction. Mechanistic studies demonstrate that DHA or DHA/ALA supplementation restores tau C-Jun N-terminal kinase (JNK) phosphorylation, total glycogen synthase kinase 3 beta (GSK3β), and attenuates hyperactivation. This restoration is accompanied by an increase in MAP 1B, dephosphorylated (active) MAP 2, and acetylated α-microtubule protein, indicating improved microtubule stability and the maintenance of active compensatory MAP (Ma et al., 2014). A transcriptomic and phosphoproteomic study of the cortical brain of tau knockout mice reveals reduced anxiety-like behavior and lower expression of fear induced by aversive conditioning, while recognition memory remains unchanged compared to wild-type mice. Analysis of cortical transcriptomic data shows transcriptional differences in multiple genes related to synaptic structure, neuronal cytoskeleton, and transport in tau knockout mice. RT-qPCR confirms increased mRNA levels of col6a4, gabrq, gad1, grm5, grip2, map 2, rab8a, tubb3, and wnt16 in tau knockout mice, as well as the absence of map 1a, compared to wild-type mice. Some proteins are validated using Western blot analysis to confirm mRNA expression changes. Map 1a mRNA and protein levels are decreased, while β-tubulin III and GAD1 protein levels are reduced in tau knockout mice (Andrés-Benito et al., 2023). Acute knockdown of tau in the adult hippocampus significantly impairs motor coordination and spatial memory, which can be reversed by restoring tau expression. In summary, tau plays a redundant role in axons; however, its physiological function becomes particularly relevant when axonal buffering capacity is reduced, especially in individuals with tau gene mutations.

## 3. The axonal milieu and tau aggregation

Axons serve as natural sites with high concentrations of tau protein. Within axons, tau can undergo modifications such as phosphorylation, which is believed to increase its propensity for aggregation. The complex environment within axons may present propitious circumstances for tau aggregation and propagation.

It is important to note that despite the significance of phosphorylated tau in tauopathies, the relationship between tau seeding activity, phosphorylated tau pathology, and neurodegenerative progression remains unclear. Studies conducted on transgenic mice expressing inducible P301L tau have shown that when the mutant tau gene is switched off, memory improves and neuronal loss halts, despite the continued accumulation of tangle burden (Santacruz et al., 2005). Another study suggested that neurons harboring tangles appear to survive in mice expressing P301L tau, despite evident disruption of the affected neurons’ membrane (de Calignon et al., 2009). In terms of the initial sites of tau pathology, some studies have indicated the locus coeruleus (LC) as the earliest region showing phosphorylated tau signals (Braak et al., 2011), while others suggest that pathology initiates in the transentorhinal/entorhinal cortex (TRE/EC) (Braak et al., 2006). Utilizing a cell-based biosensor assay to quantify tau seeding activity in fixed human tissue (Kaufman et al., 2017) and staining adjacent sections for phosphorylated tau, Kaufman et al. examined four brain regions across different disease stages in a sample of 247 individuals. They observed the earliest and most robust seeding activity in the TRE/EC, while LC exhibited uniform seeding activity only in the late stages of neurofibrillary tangle (NFT) formation (Kaufman et al., 2018). A study observed a maturation sequence of AD-associated tau aggregates, starting with the appearance of IC and IN-tau, followed by the formation of pre-tangles that displayed pT231-tau, pS396/pS404-tau, and pS202/pT205-tau, then MC1 conformational tau, and finally the formation of Gallyas-positive NFTs. Since cases classified as non-AD [Braak NFT stage <I (including a-1b)] already showed IC and IN-tau, the results suggest that these lesions are a prerequisite for AD development (Aragão Gomes et al., 2021). In chronic traumatic encephalopathy (CTE), a neurodegenerative tauopathy, tau seeding was observed in the late stages of the disease (mainly stages III and IV). This seeding was anatomically less widespread than AT8-positive inclusions, which were relatively ubiquitous. Seeding was specifically detected in the limbic system (amygdala, thalamus, basal ganglia), potentially accounting for the predominant cognitive and behavioral impairments seen in CTE (Kaufman et al., 2021). Furthermore, the impact of Aβ on tau propagation may have been overestimated (Pooler et al., 2015). A study showed that PART patients had similar progression and seeding activity patterns across all neuropathological stages to AD patients despite having less Aβ deposition (Kaufman et al., 2018), which contrasts sharply with the higher seeding activity reported in plaque cases (Bennett et al., 2017), suggesting that the phosphorylated tau spread in early AD progression occurs in an Aβ-dependent or -independent manner. In summary, these studies suggest that persistent phosphorylated tau may underlie neurodegeneration, and the different forms of tau protein may contribute to the molecular basis of distinct tau protein “strains, “leading to the wide clinical and neuropathological heterogeneity observed in tauopathies (Sanders et al., 2014). Recent applications of cryo-electron microscopy have revealed structural differences in brain-derived tau protofibrils, further highlighting the complexity of tau pathology (Shi et al., 2021). The distinction between tau phosphorylation spread and seeding activity suggests a potential transition between the two, although the exact mechanism of this transition remains unknown.

A recent significant discovery in the field of tau aggregation is the role of liquid–liquid phase separation (LLPS) in tau assembly. LLPS has been observed in various proteins with different functions under *in vitro* and physiological conditions, including synaptic structural proteins like synGAP and PSD-95 (Zeng et al., 2016), synaptotagmin-1 (Milovanovic et al., 2018), and the tumor suppressor SPOP (Bouchard et al., 2018). LLPS-based assembly has also been observed in nucleoli (Berry et al., 2015), paraspeckles (Hennig et al., 2015), and heterochromatin (Larson et al., 2017). It has been discovered that tau can nucleate microtubules *in vitro*, and this process involves liquid-phase tau (Hernández-Vega et al., 2017). Non-aggregated tubulin co-localizes with preformed tau liquid droplets, reaching the critical concentration for microtubule nucleation. Microtubules start growing bidirectionally from tau condensates a few minutes after the addition of GTP, and the growing microtubules remain “coated” by the liquid-phase tau. This mechanism of microtubule nucleation may be important for local non-centrosomal microtubule growth and organization in axons, particularly in situations where positioning is required, such as after injury or during axonal growth and branching (Hernández-Vega et al., 2017). Further investigation of the association between *in vitro* liquid tau phase and preformed microtubules revealed the formation of small local tau condensates on microtubules with very little tau concentration (approximately 0.5 nM)(Tan et al., 2019). A similar heterogeneous “coating” of tau on microtubules has also been observed in mouse brains (Dennissen et al., 2016). Interestingly, these local dense condensates appear to preferentially form in regions of high microtubule curvature, suggesting a potential mechanism for repairing or stabilizing microtubules during forced bending or axonal branching.

An interesting aspect of tau is its phosphorylation, which can significantly impact its liquid–liquid phase separation (LLPS) behavior. Tau undergoes various post-translational modifications, including phosphorylation, acetylation, ubiquitination, sumoylation, methylation, glycation, glycosylation, polyamination, nitration, isomerization, and oxidation (Guo et al., 2017). These modifications not only affect tau’s microtubule binding and aggregation properties but also its condensation behavior. For instance, mild phosphorylation by the kinase MARK2 at four sites in the microtubule-binding region (MTBR) catalyzes tau dissociation from microtubules (Matenia and Mandelkow, 2009), leading to condensation at tau concentrations of 1-2 μM (Ambadipudi et al., 2017; Wegmann et al., 2018). Another study demonstrated that cyclin-dependent kinase 2 (Cdk2) phosphorylation of tau at Alzheimer’s disease-associated sites accelerated condensation (Sang et al., 2022).

In addition to phosphorylation, RNA has been identified to have a significant role in tau nucleation. Notably, tau binds to tRNA, forming a phase state in living cells. This phase state maintains a charge balance of approximately 1: 1 between protein and nucleic acid components. The LLPS process of tau and RNA is directly influenced by salt concentration and temperature, suggesting regulation by electrostatic interactions and changes in entropy. Despite the high protein concentration within the condensate phase, tau retains its ability to tumble and diffuse through the droplet interior. Local protein packing, conformation, and dynamics of tau in the condensate phase are comparable to tau in dilute solution. In contrast, tau populations induced to aggregate by heparin complexation exhibit drastic changes in local conformation and irreversible aggregation. However, long-term residence in the droplet state eventually leads to the appearance of detectable β-sheet structures, as observed through thioflavin-T assays (Zhang et al., 2017). Another study revealed the molecular characteristics of the phase-separated state formed by peptides rich in lysine and arginine, and that the lysine-rich protein tau sequences undergo complex coalescence with RNA and associate with stress granules (SG) s (Ukmar-Godec et al., 2019). In summary, tau LLPS may have physiological significance in the complex molecular environment of axons, and also provide a condition for tau abnormal aggregation.

## 4. The bidirectional relationship between axonal injury and the pathology of Aβ and tau

Axonal dystrophy is a significant pathological change observed in the early stages of AD (Koffie et al., 2011). Both Aβ and tau have been implicated in causing damage at multiple sites along the axon, including the axon terminal, axon midsection, and axon initial segment (AIS). It is worth noting that Aβ and tau are physiological proteins of the axon, and the axon itself plays a crucial role in regulating the production and modification of Aβ and tau. Research on mouse and human neurons has shown that elevated APP levels can alter the structure of the AIS, which is responsible for initiating action potentials. The length of the AIS shortens and moves away from the cell body in response to increased APP levels. This effect is cell-autonomous, meaning it is dependent on the affected neuron itself, and exogenous Aβ, whether fibrillar or oligomeric, has no impact on the AIS structure (Ma et al., 2023). The axonal transport of tau is regulated by the axon initial segment, and the transport process is hindered by phosphorylated tau (p-tau) (Li et al., 2011). Additionally, acetylated tau has been found to impair the cytoskeletal barrier of the axon initial segment, leading to alterations in tau’s axonal localization (Sohn et al., 2016). Studies investigating the FTD-causing V337M tau mutation have revealed its impact on activity-dependent plasticity of the cytoskeleton in the axon initial segment, with abnormal accumulation of end-binding protein 3 (EB3) in the submembranous region of the AIS compromising its plasticity. These findings enhance the understanding of how tau mutations associated with FTD disrupt neuronal cytoskeletal components, leading to network dysfunction (Sohn et al., 2019). Moreover, hyperphosphorylated tau has been shown to induce a more depolarized threshold for action potential initiation and reduced firing of hippocampal CA1 neurons. These effects can be rescued by suppressing tau expression. Studies conducted on mutant and primary hippocampal neuronal cultures have demonstrated that the reduction in neuronal excitability is attributed to a downward relocation of the axon caused by phosphorylation-induced microtubule-dependent repositioning of the AIS (Hatch et al., 2017). The cleaved form of tau protein by caspase-3 (TauC3) exhibits significantly reduced dynamic interactions with microtubules. Single-molecule tracking studies have confirmed that TauC3 has longer dwell times on axonal microtubules. This decreased dynamic interaction is associated with impaired mitochondrial transport, reduced synthetic capacity of APP vesicles, and region-specific dendritic atrophy in hippocampal CA1 neurons (Conze et al., 2022).

Axonal transport function plays a role in Aβ production. Retrograde transport, mediated by dynein-Snapin, regulates the transport of β-secretase (BACE1) and the processing of presynaptic APP in axons (Ye et al., 2017). Genetic ablation of this retrograde transport pathway leads to increased retention of BACE1 in late endosomes and enhanced APP processing at presynaptic sites in mice. Conversely, overexpression of Snapin promotes BACE1 transport and reduces its accumulation at synapses by facilitating its removal from distal axons and presynaptic terminals in AD (Ye et al., 2017). Recent studies have identified myelin dysfunction and demyelination injury as drivers of amyloid deposition in AD mouse models. Mechanistically, myelin dysfunction leads to the accumulation of Aβ-generating machinery within axonal swellings and increases cortical APP cleavage. Age-dependent structural defects in myelin directly or indirectly promote the formation of Aβ plaques (Depp et al., 2023). These findings highlight the complex interplay between axonal transport, protein cleavage, and myelin dysfunction in AD pathogenesis, further emphasizing the importance of understanding the molecular mechanisms underlying axonal abnormalities in the disease. In conclusion, APP and tau may be abnormally modified and aggravate axonal damage when MTs are broken or other axonal injuries occur.

## 5. The role of axonal injury plays in the changes of structure in neuron

### 5.1. Axonal injury and synaptic dysfunction

Synaptic dysfunction and loss are key features of AD, and synaptic loss is closely associated with cognitive decline in the disease (Mecca et al., 2022). The spread of soluble Aβ and tau proteins through synapses is considered an important mechanism of disease progression (Colom-Cadena et al., 2023; Tzioras et al., 2023). Moreover, there is synaptic specificity in AD, indicating that intrinsic properties of synapses contribute to the disease (Griffiths and Grant, 2022). Recent reviews have nicely summarized the effects of Aβ and tau on presynaptic terminals (John and Reddy, 2021; Tzioras et al., 2023). Nevertheless, normal axonal function is an important guarantee for maintaining synaptic plasticity (Guedes-Dias and Holzbaur, 2019). Axonal transport, as the upstream process in neural connections, plays a crucial role in regulating synaptic plasticity. Local regulation of cytoskeletal dynamics and organelle transport facilitates synaptic-specific delivery and plasticity, highlighting the importance of axonal transport in synaptic function (Aiken and Holzbaur, 2021). Studies on developing mouse neuromuscular junctions have revealed that axonal branch loss is mediated by branch-specific microtubule severing, leading to dismantling of the microtubule cytoskeleton and loss of axonal transport in the affected branch. Pharmacological stabilization of microtubules can delay the elimination of neuromuscular synapses. The microtubule severing enzyme spastin is implicated in this branch-specific cytoskeletal dismantling, and its dysfunction is associated with certain forms of upper motor neuron diseases (Brill et al., 2016). Similar mechanisms involving microtubule severing enzymes may be involved in various motor neuron diseases and neurodegenerative diseases, including AD, which exhibit axonal damage. In summary, axonal transport has a direct impact on synaptic plasticity and influences the intrinsic characteristics of synapses, which are crucial in the progression of AD and other neurodegenerative diseases. Understanding the role of axonal transport in synaptic function and its implications for disease pathology is an important area of research.

### 5.2. Axonal injury and neuronal death

The relationship between axonal injury and neurodegeneration is still a topic of ongoing research and debate. One major question is whether the axonal injury is merely a consequence of neurodegeneration or if it plays a key role in initiating the disease process (Berth and Lloyd, 2023).

Wallerian degeneration describes the sequential degeneration of the axon after axonal injury, starting from cytoskeletal breakdown and ending with fragmentation and loss of the detached distal axon (Conforti et al., 2014; Zhang et al., 2021). Wallerian degeneration and axonal degeneration in neurodegenerative diseases share similarities, including cytoskeletal breakdown (Cowan et al., 2010), axonal transport disruption (Tang-Schomer et al., 2012), mitochondrial morphological change (Park et al., 2013b), and in the central nervous system (CNS), axonal swellings (Wirths et al., 2007; Tang-Schomer et al., 2012). Additionally, compelling experimental and pathological studies suggest that neurons in AD exhibit a dying-back pattern of neurodegeneration, where axon terminals and axons progressively degenerate toward the neuronal cell body (Bell and Claudio Cuello, 2006; Gilley et al., 2011; Nishioka et al., 2019; Salvadores et al., 2020). This indicates that they share similar processes. It is important to note that early axonal pathology in AD may still retain some physiological function, but it could serve as a precursor to neuronal death. Overall, the evidence suggests that axonal degeneration is a significant component of neurodegenerative diseases and may be a prerequisite for neuronal death. However, further research is needed to fully understand the causal relationship between axonal transport disruption and the initiation and progression of neurodegenerative diseases.

## 6. Axonal injury occurs in the early stage of AD

White matter aging is a significant component of brain aging and has implications for cognitive function. Regional white matter integrity, rather than cortical thickness, has been found to be positively correlated with cognitive functions such as episodic memory, semantic memory, and frontal executive function. This highlights the importance of white matter in brain aging (Ziegler et al., 2010). Functional connectivity studies have also emphasized the role of white matter in brain aging and cognitive function (Damoiseaux, 2017).

A single-cell analysis study conducted on postmortem brains from individuals with sAD revealed pathological transcriptional features in various cell types, including neurons, neuroglia, and 40 distinct cell subpopulations. Notably, myelination-associated genes were found to be disrupted not only in oligodendrocytes and oligodendrocyte progenitor cells (OPCs) but also in most major cell types, suggesting a primary regulatory response aimed at maintaining proper myelin integrity (Mathys et al., 2019). In the context of Alzheimer’s disease, studies have shown that AD patients exhibit lower frontal white matter volume compared to age-matched healthy elderly individuals, even when the gray-white matter volume ratio changes insignificantly (Salat et al., 1999). Recent research by Prescott et al. investigated early brain imaging changes in individuals with autosomal dominant inherited Alzheimer’s disease. They discovered that mutation carriers had lower connectivity within white matter structures of the frontal–parietal control network (default mode network, frontal–parietal control network, and ventral attention network) compared to non-carriers, even after accounting for various factors such as age, gender, education level, apolipoprotein E4 allele status, white matter volume, Mini-Mental State Examination score, and estimated years to dementia onset. This suggests that mechanisms beyond amyloid pathology contribute to white matter alterations preceding cognitive decline in autosomal dominant inherited AD (Prescott et al., 2022). Furthermore, in AD patients, significant white matter loss, both microstructurally and macrostructurally, has been observed independently of amyloid accumulation, particularly in fiber pathways associated with default mode network nodes. Reduced density and cross-sectional area of white matter fibers in the posterior cingulate have been found to be independent of high amyloid accumulation (Mito et al., 2018). Interestingly, white matter hyperintensities (WMHs), which are imaging alterations characterized by brain white matter rarefaction and often associated with demyelination, edema, and reactive gliosis (Grinberg and Thal, 2010), were associated with a twofold increased likelihood of Aβ positivity after the age of 65 (Habes et al., 2021). Moreover, high WMHs in axons were linked to cognitive impairment in semantic fluency and executive function, with this relationship being mediated by temporal–parietal atrophy rather than metabolic factors (Ottoy et al., 2023). A meta-analysis incorporating 19 prospective studies with 40,040 participants demonstrated that baseline WMHs increased the risk of cognitive impairment, all-cause dementia, and Alzheimer’s disease by 14 and 25%, respectively (Hu et al., 2021). Furthermore, white matter hyperintensities contribute to the pattern of brain atrophy associated with Alzheimer’s disease (Ma et al., 2022) and cognitively normal APOE4 carriers also show white matter impairment. A study that used diffusion tensor imaging to investigate the potential effect of APOE polymorphism on WM structure in healthy young (20–35 years) and elderly (50–78 years) individuals showed that, compared to non-carriers, carriers of the APOE4 allele exhibited widespread reductions in fractional anisotropy and increased mean diffusivity values. Interestingly, no significant interaction between genotype and age was observed, indicating that the WM structural differences between APOE4 carriers and non-carriers did not change significantly with age (Heise et al., 2011). Another study involving 4,527 participants showed that around 25% of the cognitive impact of APOE4 was mediated by white matter lesion volume and total brain tissue volume, and approximately 9% of the total effect of APOE4 carriership on cognition was mediated by white matter lesion volume (Ma et al., 2022). Considering that APOE4 is the second-largest risk factor for AD after aging (Serrano-Pozo et al., 2021), these structural differences in white matter may contribute to the disease’s development.

Additionally, tau pathology has also been associated with white matter impairment. A study demonstrated that white matter decline was related to early tau accumulation, and further WM decline might reflect the propagation of tau in the later stages of AD (Strain et al., 2018). Moreover, white matter damage is highly associated with small vessel disease (Wardlaw et al., 2019), and the high comorbidity of small vessel disease with AD further underscores the importance of white matter damage. In conditions such as CET, where repetitive head impacts are involved, white matter rarefaction, arteriosclerosis, and the presence of neurofibrillary tangles in the deep layers of the frontal cortex were independently associated with dementia (Alosco et al., 2019).

## 7. The challenges axon faces during aging

In AD and aging, axons face multiple challenges related to energy metabolism, reactive oxygen species (ROS), and other factors. Here we mainly discuss the possible mechanisms in relation to the known AD risk genes.

### 7.1. Mitochondria, glucose metabolism, and reactive oxygen species

The brain, despite representing only 2% of the average body weight, consumes a substantial amount of glucose and oxygen, accounting for approximately 25 and 20% of the body’s total usage, respectively, to meet its metabolic demands (Neth and Craft, 2017). In fact, about 80% of the total energy is consumed by the brain to maintain axonal and synaptic function (Small et al., 2000). Central metabolic dysfunction is a recognized feature of AD, as evidenced by reduced brain glucose metabolism, which can be observed decades before the onset of AD symptoms (Small et al., 2000). Recent studies have emphasized the crucial role of insulin in brain and peripheral metabolism, as well as other functions (Neth and Craft, 2017). ROS are byproducts of cellular metabolism, including mitochondrial respiration. In aging and AD, an imbalance between ROS production and antioxidant defenses leads to increased oxidative stress (Ahmad et al., 2017). ROS can cause damage to cellular components, including axonal structures and transport machinery, thereby impairing axonal transport and contributing to neurodegeneration. Macrophage-derived reactive oxygen and nitrogen species (ROS and RNS) can initiate axonal degeneration by inducing mitochondrial pathology. In fact, neutralization of ROS and RNS has been shown to rescue degenerating axons (Nikić et al., 2011). Another study revealed that the expression of NOX2, an enzyme that produces ROS, in microglia was associated with axonal initial segment (AIS) disruption in the presence of acute microglial inflammation (Benusa et al., 2017). Furthermore, exogenous hydrogen peroxide has been found to rapidly inhibit axonal transport, primarily affecting mitochondrial transport before Golgi-derived vesicle transport. Anterograde vesicle transport is more susceptible to peroxide inhibition than retrograde transport, indicating a differential effect of ROS on axonal transport (Fang et al., 2012).

In addition, studies using diffusion tensor imaging (DTI) have demonstrated that white matter microstructure and connectivity undergo widespread changes associated with type 2 diabetes mellitus (T2DM), which are significantly correlated with cognitive impairment (Moran et al., 2017). In fact, axonal transport abnormalities have been implicated in diabetic peripheral neuropathy for nearly 30 years (Medori et al., 1985). DM may cause alterations in cytoskeletal components, such as actin, neurofilaments, and tubulin, which are essential for normal intracellular (axonal) transport, either by direct glycation under hyperglycemic conditions or due to impaired gene expression (Cullum et al., 1991; Brown et al., 1992; Mohiuddin and Tomlinson, 1997). For example, tubulin glycation in DM impairs polymerization and alters microtubule dynamics, which are crucial for the transport of intracellular macromolecules and organelles (Williams et al., 1982).

Mitochondrial dysfunction can contribute to axonal damage. Myelin damage is one of the manifestations of axonal injury. It is worth noting that axonal degeneration can occur even in the presence of apparently normal myelin. Previous studies have demonstrated that axonal pathology preceding axonal degeneration includes impairment of axonal transport and altered formation of axonal spheroids in mice deficient in PLP (proteolipid protein) and CNP (2′,3′-cyclic nucleotide 3′-phosphodiesterase) (Griffiths et al., 1998; Lappe-Siefke et al., 2003), as well as reduced neurofilament phosphorylation and smaller axonal diameter in MAG (myelin-associated glycoprotein) deficient mice (Yin et al., 1998). These lesions are particularly prominent in the paranodal regions of the internodes, where communication between myelin and axons is likely to occur. More recently, a study using a mutant mouse model, where the central myelin protein PLP was replaced by the peripheral myelin protein P0, revealed a series of changes in axonal degeneration. This study found that mitochondrial pathology and degeneration were prominent in juxtaparanodal axoplasm at 1 month of age, while MT pathology and paranodal axon spheroids were observed at 6 months. These abnormalities collectively indicate that abnormal mitochondrial energy metabolism precedes axonal degeneration (Yin et al., 2016). Enhancing axonal mitochondrial transport through the deletion of syntaphilin (Snph) has been shown to restore injury-induced mitochondrial depolarization. Mice lacking Snph exhibit enhanced regeneration of the corticospinal tract (CST) after spinal cord lesion, accelerated regeneration of monoaminergic axons across the transection gap, and increased compensatory sprouting of uninjured CST. Importantly, the regenerating CST axons form functional synapses and contribute to motor function recovery. The administration of the bioenergetic compound creatine enhances the CST regenerative capacity in Snph knockout mice (Han et al., 2020).

The intronic GGGGCC repeat expansion in C9ORF72 is the most common genetic cause of amyotrophic lateral sclerosis (ALS) and FTD. One of the main pathological features of the repeat expansion is the accumulation of proteins containing dipeptide repeat sequences (DPRs) (Tang et al., 2020). Axonal injury is a common characteristic of ALS (Baldwin et al., 2016; Mehta et al., 2019). A study revealed that C9ORF72 plays a significant role in motor neurons by interacting with endosomes, facilitating normal vesicle transport, and contributing to lysosomal biogenesis. Modulating vesicle transport can rescue neurodegeneration caused by C9ORF72 repeat expansion (Shi et al., 2018). Additionally, Poly (GR), one of the DPRs, preferentially binds to the mitochondrial complex V component ATP5A1, enhancing its ubiquitination and degradation (Choi et al., 2019). Another study demonstrated that enhancing bioenergetics in motor neurons alone is sufficient to restore axonal homeostasis (Mehta et al., 2021). Furthermore, inhibition of RIPK1 prevented mitochondrial fragmentation *in vitro* and *in vivo*, and inhibiting mitochondrial fission resulted in reduced loss of injured axons (Arrázola et al., 2019).

### 7.2. Autophagy

Autophagy defects rather than excessive autophagy induction have been associated with axonal and dendritic dystrophy, leading to the loss of synaptic integrity in various neurodegenerative conditions (Frake et al., 2015; Menzies et al., 2017). A study demonstrated that the induction of autophagy promotes neuronal outgrowth, mitigates the inhibitory effects of non-permissive substrate myelin, and reduces the formation of retraction bulbs in cultured cortical neurons following axonal injury. Notably, autophagy induction stabilizes microtubules by degrading SCG10, which is a protein known to destabilize microtubules in neurons. In mice with spinal cord injuries, local administration of the autophagy inducer peptide Tat-beclin1 significantly attenuated axonal retraction in both the dorsal columns and the corticospinal tract at the injury site. Furthermore, Tat-beclin1 administration facilitated the regeneration of descending axons during long-term observation (He et al., 2016). In Tauopathy, impaired retrograde transport of autophagosomes in axons was observed due to the impairment of the dynein-dynactin complex (Butzlaff et al., 2015). Autophagy is typically inhibited by the mammalian target of rapamycin (mTOR), an evolutionarily conserved regulator of cell growth that integrates signals from growth factors, nutrients, and cellular stress by upregulating protein synthesis (Kim and Guan, 2019). Notably, pharmacological studies using autophagy inducers such as trehalose and dimethyl sulfoxide, or mTOR inhibitors like rapamycin, bexarotene, and lactacystin in animal models of human tauopathy support the notion that autophagy involvement in Tauopathy may confer beneficial effects (Congdon et al., 2012; Schaeffer and Goedert, 2012).

### 7.3. Lipid turnover and myelination

Myelin, the insulating sheath around nerve fibers, is primarily composed of lipids (approximately 70%) and proteins (approximately 30%). It is known for its high content of cholesterol and cerebrosides, contributing to its unique composition (Chrast et al., 2011). Recent studies have revealed that myelin turnover in the CNS is a dynamic process. Analysis using radiocarbon dating from nuclear bomb tests has shown that myelin is exchanged at a high rate, while the population of oligodendrocytes, the cells responsible for myelin production, remains remarkably stable in white matter, with an annual exchange rate of approximately 1/300 oligodendrocytes (Yeung et al., 2014). The Quaking (Qki) protein has been identified as a major regulator of myelin lipid homeostasis. Depletion of Qki specifically in oligodendrocytes results in rapid demyelination within 1 week and progressive neurological deficits in adult mice, without affecting oligodendrocyte survival (Zhou et al., 2020). Qki depletion leads to a significant reduction in myelin lipids, particularly monounsaturated fatty acids, and very long chain fatty acids, while major myelin proteins remain intact. Interestingly, a high-fat diet has been shown to ameliorate the demyelination phenotype in Qki-depleted mice, suggesting a potential role for dietary lipids in modulating myelin integrity (Zhou et al., 2020). Furthermore, studies have demonstrated the importance of lipid metabolism in axonal regeneration. For example, axonal regeneration induced by the deletion of lipin1, a key enzyme involved in triglyceride synthesis, requires triglyceride hydrolysis and phospholipid synthesis. Deleting neuronal diacylglycerol acyltransferases (DGATs), enzymes responsible for triglyceride synthesis, and enhancing phospholipid synthesis through the Kennedy pathway promotes axonal regeneration (Yang et al., 2020). These findings highlight the crucial role of triglycerides and phospholipids in supporting axonal regrowth.

Overall, lipid turnover and homeostasis play essential roles in myelination, maintenance of myelin integrity, and axonal regeneration. Understanding the molecular mechanisms underlying lipid metabolism in the context of myelin and axonal function could have implications for developing therapeutic strategies targeting demyelinating diseases and promoting axonal repair.

## 8. The effects of genetic risks of AD on axon

### 8.1. The effects of APOE on axon

Human APOE is a 34 kDa glycoprotein with three major allele variants: APOE2, APOE3, and APOE4. Among these, APOE4 is associated with an increased risk of AD, while APOE2 is linked to a reduced risk compared to the common APOE3 allele (Yamazaki et al., 2019). Numerous large-scale genome-wide association studies and meta-analyses have consistently identified the APOE4 allele as the strongest genetic risk factor for sAD, whereas the APOE2 allele confers the strongest genetic protective effect (Serrano-Pozo et al., 2021). Interestingly, APOE isoforms, being apolipoproteins, influence APOE-lipid interactions, which play a role in the transport of lipids to neurons. APOE4 exhibits a loss-of-function effect, as it is less efficient than APOE2 or APOE3 in lipid transportation to neurons [summarized in (Yamazaki et al., 2019)].

The transport of lipids mediated by APOE plays a crucial role in maintaining axonal stability. Cholesterol, a significant component of membrane bilayers, is known to affect membrane integrity and is associated with the risk of neurodegenerative diseases (Arenas et al., 2017; Dai et al., 2021). Neurons and the brain experience an age-dependent reduction in cholesterol content (Colin et al., 2016), leading to impaired intracellular signaling and synaptic plasticity (Palomer et al., 2016; Martín-Segura et al., 2019). This decline in membrane fluidity, coupled with changes in receptor conformation due to reduced cholesterol levels, contributes to reduced synaptic plasticity and defects in insulin signaling induced by glutamate receptor activation and receptor tyrosine kinase (RTK) signaling (Martín-Segura et al., 2019). Recent studies have also demonstrated that cholesterol depletion from the membrane enhances tau entry and intracellular tau aggregation (Tuck et al., 2022). A similar effect was observed following the depletion of Niemann-Pick C1 protein, which disrupts cholesterol transport and leads to Niemann-Pick disease (Tuck et al., 2022). Interestingly, elevated cholesterol on the lysosomal membrane in Niemann-Pick disease type C (NPC) sequesters motor adaptors that are transported by axonal lysosomes. This aberration impairs anterograde lysosome transport to distal axons, disrupting axonal autophagosome-lysosome fusion during retrograde transport. Altering the lipid composition of the membrane impairs axonal lysosome transport and localization, while reducing lysosomal lipid levels rescues lysosome transport to NPC axons, thereby reducing axonal autophagy stress in the early stages of NPC disease (Roney et al., 2021). Cerebrospinal fluid cholesterol efflux capacity is impaired in individuals homozygous for the APOE4 allele (Yassine et al., 2016). APOE4 significantly affects signaling pathways associated with cholesterol homeostasis and transport. Histological and lipidomic analyses of postmortem human brains, induced pluripotent stem cell-derived cells, and targeted replacement mice have revealed abnormal cholesterol accumulation in oligodendrocytes, the cells responsible for insulating and promoting neuronal electrical activity. This altered cholesterol localization in APOE4 brains corresponds to reduced myelin formation. Pharmacological enhancement of cholesterol transport has been shown to increase axonal myelination and improve learning and memory in APOE4 mice (Blanchard et al., 2022).

Interestingly, APOE4 derived from astrocytes and neurons may have different roles. Astrocytic APOE4 induces the strongest neuronal firing, while neuronal APOE3 promotes the most active and efficient neuronal activity (Konings et al., 2021). Importantly, the removal of astrocytic APOE4 has been found to reduce tau-induced synaptic loss and microglial phagocytosis of synaptic elements, suggesting a crucial role of astrocytic APOE4 in synaptic degeneration (Wang et al., 2021). Similarly, selectively reducing astrocytic APOE3 and APOE4 resulted in a significant decrease in Aβ plaque deposition and plaque density (Mahan et al., 2022). Additionally, in patients with traumatic brain injury (TBI), axonal injury leads to long-term neurological deficits. It has been reported that APOE4 activates intracellular adaptor protein Disabled-1 (Dab1) phosphorylation through interactions with APOE4 receptors. The Dab1 pathway functions as a regulator of axonal growth and growth cone formation in the brain (Huang et al., 2020). In summary, these studies emphasize the crucial role of APOE4 in regulating lipid balance in the central nervous system, as well as its involvement in AD pathology.

### 8.2. The effects of TREM2 on axon

TREM2 (triggering receptor expressed on myeloid cells 2) is a transmembrane receptor belonging to the immunoglobulin superfamily (Deczkowska et al., 2020). It interacts with various anionic ligands, including bacterial products, DNA, lipoproteins, and phospholipids, either in their free form or bound to the membrane (Deczkowska et al., 2020). Notably, homozygous functional loss mutations of TREM2 and DAP12 lead to Nasu-Hakola disease (NHD), which is characterized by progressive presenile dementia and cystic lesions associated with recurrent fractures (Klünemann et al., 2005). Genome-wide association studies (GWAS) analyzing AD patients have revealed that rare variants in TREM2, such as Arg47 → His (R47H) and the more common R62H variant, increase the risk of AD (Sims et al., 2017). These variants affect the stability of TREM2, impair its phagocytic ability, and alter its affinity for APOE4, clusterin (ApoJ), low-density lipoprotein, and Aβ (Hansen et al., 2018). Importantly, TREM2 is essential for microglial activation (Ulland et al., 2017). Knockout or knockdown of TREM2 in AD animal models has been shown to reduce the microglial barrier around plaques, decrease plaque compaction, lower APOE4 levels in APP mice, and increase the presence of dystrophic neurites surrounding plaques (Jay et al., 2015; Wang et al., 2016; Jay et al., 2017; Parhizkar et al., 2019).

TREM2 serves multiple roles *in vivo*, which have been reviewed recently (Deczkowska et al., 2020). Additionally, TREM2 plays a crucial role in clearing axonal fragments. A study demonstrated that cholesterol esterification in microglia is an adaptive response to myelin fragment uptake, leading to lipid droplet formation during demyelinating injury. Impaired lipid droplet biogenesis results in unresolved innate immune response and failed regeneration. In mice deficient in triggering receptor expressed on myeloid cells 2 (TREM2), excessive cholesterol exposure cannot be properly managed, resulting in reduced lipid droplet formation and endoplasmic reticulum (ER) stress. Alleviating ER stress in TREM2-deficient mice restores lipid droplet biogenesis and resolves the innate immune response (Gouna et al., 2021). Moreover, in a focal demyelination model in the brain, TREM2 knockout mice showed persistent demyelination for over 6 weeks following lysolecithin injection, leading to severe neurodegeneration (Wang et al., 2023). Treatment with a novel TREM2 agonist antibody enhances microglial clearance of myelin debris through increased uptake and degradation of myelin lipids in a CNS demyelination cuprizone model. Another study demonstrates that an agonistic monoclonal antibody targeting TREM2, AL002c, promotes microglial proliferation, reduces fibrillar plaques and dystrophic neurites, and alleviates microglial inflammatory response in a mouse AD model (Wang et al., 2020). Importantly, antibody-dependent TREM2 activation on microglia increases the density of oligodendrocyte precursors in the demyelinated area, promoting the formation of mature oligodendrocytes, thus enhancing myelin regeneration and maintaining axonal integrity (Cignarella et al., 2020).

TREM2 (triggering receptor expressed on myeloid cells 2) contains an ectodomain that can be cleaved by sheddases, leading to the release of soluble TREM2 (sTREM2) into the extracellular space. The exact endogenous function of sTREM2 is not fully understood, but it is hypothesized that sTREM2 shedding occurs following ligand binding, thus blocking downstream intracellular signaling. In this context, continuous maturation and transport of newly synthesized TREM2 protein to the cell surface are required for sustained receptor activity. The levels of sTREM2, measured in cerebrospinal fluid or plasma, may reflect the extent of TREM2 receptor binding to its ligands (soluble or cell-bound), the shedding of sTREM2, and the flux of newly synthesized TREM2 protein through the transport pathway. Additionally, to some extent, sTREM2 levels may reflect the activation state of microglia (Filipello et al., 2022). Blocking the hydrolytic shedding of TREM2 enhances downstream receptor signaling and function, including microglial phagocytic activity (Schlepckow et al., 2020). In conditions of increased microglial activation, such as in inflammatory neurological diseases, there is evidence of upregulated the expression of TREM2 and elevated levels of sTREM2 in cerebrospinal fluid (Diaz-Lucena et al., 2021). Importantly, according to the Braak staging system in AD, microglial activation correlates with the progression of tau pathology (Pascoal et al., 2021). A study investigating subjects across the aging and AD spectrum using PET imaging for microglial activation and tau pathology found that cerebrospinal fluid sTREM2, along with [C_11_]PBR28 PET imaging, may serve as an indicator of *in vivo* microglial activation. This microglial activation showed strong associations with tau pathology, brain atrophy, vascular white matter pathology, and cognitive impairment, supporting the relationship between sTREM2 and AD pathophysiology (Pascoal et al., 2021). Additionally, a study reported elevated levels of CSF sTREM2 in individuals with evidence of tau pathology and neurodegeneration biomarkers but not Aβ pathology, which instead showed reduced sTREM2 levels (Suárez-Calvet et al., 2019). Furthermore, recent studies have demonstrated that plasma sTREM2 is independently associated with tau-positive scans and white matter hyperintensity volume but not amyloid burden in patients with Alzheimer’s disease and cerebral amyloid angiopathy, the most common sporadic small vessel disease (Tsai et al., 2021). In summary, the TREM2 axis plays an important role in regulating microglial function and is a necessary signaling pathway for maintaining normal myelin and axonal function.

## 9. The application of axonal microtubule stabilizers

Axonal microtubule stabilizers have long been proposed as potential therapeutic agents for tauopathies, but their promising prospects have only recently emerged due to challenges posed by the blood–brain barrier and the side effects associated with inhibiting peripheral cell division (Ballatore et al., 2012). One such microtubule stabilizer is EpoD (Epothilone D). In a groundbreaking study conducted in 2020, EpoD (also known as BMS-241027) was administered to APP/PS1 mice, demonstrating its ability to alleviate axonal/synaptic damage, dystrophic neurites, neuronal loss, and even reduce amyloid accumulation (Fernandez-Valenzuela et al., 2020). Another promising microtubule stabilizer is CNDR-51657, a drug belonging to the triazole-pyrimidine class, which binds to microtubule proteins at the same site as vincristine (Zhang et al., 2018). In an oral administration study using 9-month-old female PS19 mice, CNDR-51657 significantly alleviated microtubule defects, axonal dystrophy, and tau pathology, with no reported adverse reactions (Zhang et al., 2018). The favorable properties of good brain permeability and oral bioavailability make CNDR-51657 a promising candidate for microtubule stabilization therapy (Oukoloff et al., 2021). Nevertheless, in a double-blind randomized clinical trial, use of the microtubule stabilizer TPI-287 elicited severe hypersensitivity reactions in Alzheimer’s disease patients. Moreover, with higher dosing, clinical worsening and biomarker changes were observed in patients with progressive supranuclear palsy and corticobasal syndrome, despite the inability of this study to demonstrate binding of TPI-287 to axonal microtubules (Tsai et al., 2020). In summary, although microtubule stabilizers are anticipated to become salient pharmaceuticals for the treatment of tauopathies, further experimentation is imperative.

## 10. Conclusion

Axonal injury is believed to be one of the earliest pathological changes in AD and continues to play a role in disease progression. Recent studies have highlighted the interconnectedness of Aβ accumulation, tau aggregation, and axonal transport dysfunction, which suggests the potential for targeting axonal function as a therapeutic approach for AD and tauopathies. However, it is important to note that our hypothesis does not fully explain the specific vulnerability of neurons (Praschberger et al., 2023), an aspect that requires further investigation. Nevertheless, our review offers new insights into the etiology and treatment of AD, providing a foundation for future research in this field.

## Author contributions

LD: Writing – original draft, Writing – review & editing. ZZ: Funding acquisition, Supervision, Writing – review & editing.
